# mCherry Fusion Proteins Facilitate Production of Recombinant, Cysteine-Rich *Leptospira interrogans* Proteins in *Escherichia coli*

**DOI:** 10.21203/rs.3.rs-2931251/v1

**Published:** 2023-05-18

**Authors:** Reetika Chaurasia, Cathleen Liang, Kenneth How, Dielson S. Vieira, Joseph M. Vinetz

**Affiliations:** 1 Section of Infectious Diseases, Department of Internal Medicine, Yale School of Medicine, New Haven, CT, United States

**Keywords:** Soluble protein, mCherry, fluorescence intensity, PF07598 gene family, VM proteins

## Abstract

**Background:**

Recombinant fluorescent fusion proteins are fundamental to advancing many aspects of protein science. Such proteins are typically used to enable the visualization of functional proteins in experimental systems, particularly cell biology. An important problem in biotechnology is the production of functional, soluble proteins. Here we report the use of mCherry-fusions of soluble, cysteine-rich, *Leptospira*-secreted exotoxins in the PF07598 gene family, the so-called virulence modifying (VM) proteins.

**Results:**

The mCherry fusion proteins facilitated the production of the VM proteins (LA3490 and LA1402) by enabling the visual detection of pink colonies and following them through lysis and sequential chromatography steps. CD-spectroscopy analysis confirmed the stability and robustness of the mCherry-fusion protein, with a structure comparable to AlphaFold structural predictions. LA0591, a unique member of the PF07598 gene family that lacks N-terminal ricin B-like domains, was produced as a tagless protein that strengthens the recombinant protein production protocol. The current study provides the approaches for the synthesis of 50–125 kDa soluble, cysteine-rich, high-quality mCherry tagged or tagless fast protein liquid chromatography (FPLC)-purified protein.

**Conclusions:**

The use of mCherry-fusion proteins enables a streamlined, efficient process of protein production and qualitative and quantitative downstream analytical and functional studies. Approaches for troubleshooting and optimization were systemically evaluated to overcome difficulties in recombinant protein expression and purification, demonstrating biotechnology utility in accelerating recombinant protein production.

## Background

Life science research revolves around understanding the relationship of protein structure to function, and proper three-dimensional folding of recombinant proteins is essential for determining protein function (1, 2). *Escherichia coli* is an ideal organism for this work given its defined genome, short generation time, high yield at low cost, and convenience for producing fluorescent protein-tagged recombinant proteins (3–5). Flexibility in expression conditions to enable proper protein folding such as low-temperature (16°C) shift upon IPTG induction can be exploited in *E. coli* expression systems to enhance protein yield, limit aggregation, and produce a soluble function proteins (2, 6). Expression of cysteine-rich proteins as functional toxins can be challenging because of the disulfide bonding of heterologous over-expressed proteins, leading to the inclusion body formation (1, 7–10).

The discovery of fluorescent tags facilitates real-time monitoring of the function of proteins, nucleic acids, organelles, and organisms without cytotoxic effects (5, 11). mCherry is a broadly used monomeric (28.8 kDa) red fluorescent protein derived from the Indo-Pacific Sea anemone, *Discosoma sp*. (12). mCherry is photostable and emits light at a wavelength of 610 nm and is stable in high concentrations of urea, making it suitable as a fusion partner for downstream applications and facilitating the production of soluble, function recombinant proteins (12, 13).

We recently discovered the novel paralogous PF07598 gene family (VM proteins)—found only in group I pathogenic *Leptospira* species and expanded in most virulent *L. interrogans, L. kirschneri, and L. noguchii* species, whose genomes encode a repertoire of ≥ 12 distinct paralogs (14–16). VM proteins all have encoded signal peptides that suggest they are secreted; this has been confirmed experimentally (17, 18). Not only are VM proteins secreted exotoxins but they are comprised of *bona fide* N-terminal tandemly repeated ricin B-like lectin domains and a C-terminal DNase/toxin domain. Experimental work has shown the leptospiral VM proteins to be potential vaccine candidates (18, 19). VM protein genes are upregulated *in vivo* in the acute hamster model, consistent with the hypothesis that VM proteins are involved in mediating leptospirosis pathogenesis (14–16). The full-length VM proteins are predicted to have twelve cysteines and five disulfide bonds, and their expression as soluble, functional VM proteins has been challenging.

This study aimed to optimize the production of soluble, functional, VM protein-mCherry VM fusion proteins for real-time visualization of the expression and purification processes. The protocol is accessible, reproducible, and potentially applicable to produce bacterial exotoxins in either multi-globular or individual domains.

## Results

### Cysteine-rich multi–globular domain soluble leptospiral VM proteins

**Figure 1.**
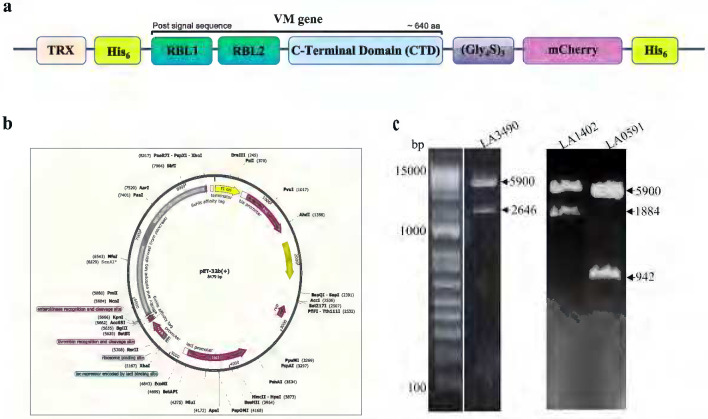
Schematic diagram of VM gene and construct map. **(a)** VM proteins are multi-globular domain proteins. The N-terminal and C-terminal were tagged with thioredoxin, and mCherry respectively, and His_6_ was tagged at both N and C- terminals. **(b)** VM gene was cloned at multiple cloning sites into pET-32b (+) vector using *XhoI* and *HindIII* restriction enzymes. **(c)** The recombinant clones LA3490 (left panel), LA1402- and LA0591 (right panel) were confirmed by restriction digestion. Arrows indicate vector (5900 bp) and inserts (*la3490*-2646 bp, *la1402*-1884 bp, and *la0591*-942 bp).

**Figure 2.**
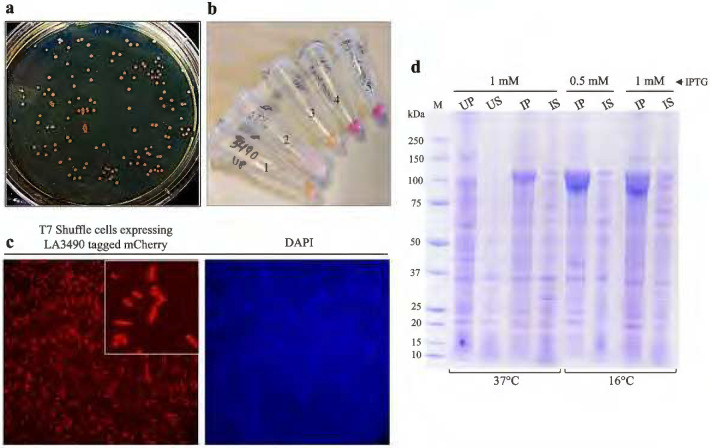
Expression and optimization of LA3490 VM protein. **(a)** The recombinant clone expressing mCherry-LA3490, shown by pink colonies. **(b)** The recombinant clone was induced with different concentrations (1mM and 0.5mM) of IPTG and temperatures (37°C and 16°C). Eppendorf tubes 1 and 3 show uninduced culture grown at 37°C and 16°C respectively. Eppendorf tubes 2, 4, and 5 show culture induced with 1mM IPTG at 37°C, 1mM IPTG at 16°C, and 0.5mM IPTG at 16°C temperature respectively. **(c)** *E. coli* expressing mCherry-LA3490 (left panel) were visualized under a fluorescence microscope (Olympus, Japan) at 40X objective, and chromosomes were stained with DAPI (right panel). **(d)** Uninduced and induced cultures were lysed with lysis buffer (details in experimental procedures) and 15 *μ*g were analyzed on 4–12% SDS-PAGE followed by Coomassie stain. Molecular weight shows in kDa. UP and US encode for uninduced pellet and uninduced supernatant. IP and IS encode for induced pellet and induced supernatant.

**Figure 3.**
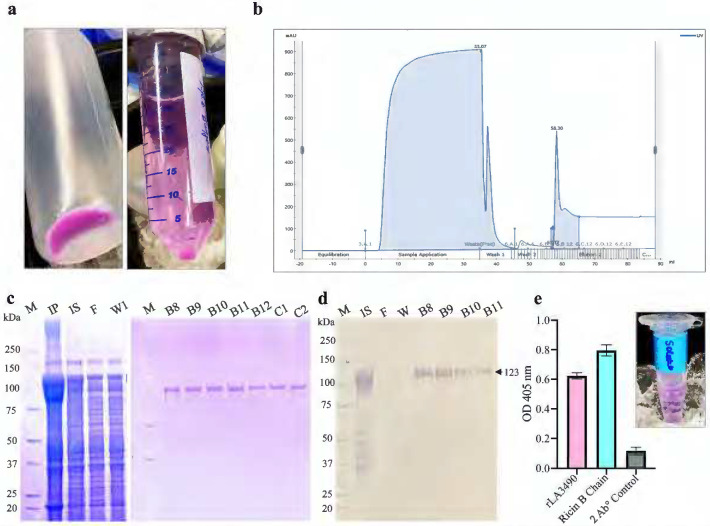
Production and functional activity of mCherry-LA3490. **(a)** mCherry-LA3490 protein visualized by pink color due to tagged mCherry fluorescent protein (left panel). The pellet was solubilized, and the supernatant was separated (right panel). **(b)** Elution peak of solubilized protein purified using AKTA pure, shown by chromatography. **(c)** Eluted fractions were analyzed by 4–12% SDS-PAGE followed by Coomassie stain. IP and IS represent induced pellet and induced supernatant. F and W1 show flow through and wash. B2-C3 are the eluate fractions. M represents the molecular weight marker. **(d)** Western blot showing the reactivity of mCherry-LA3490 with His_6_-monoclonal antibody. **(e)** The functional activity of mCherry-LA3490 was confirmed by an asialofetuin binding assay. The ricin B chain served as positive control and the secondary antibody alone served as a negative control. Soluble proteins are shown in the Eppendorf tube (top right panel).

The real-time monitoring of expression and purification of VM protein was feasible by tagging mCherry fluorescent protein, visualized by pink color (Fig. 1–3) (20, 21). Efficiency mCherry-fusion protein expression was seen with 1 mM IPTG at 16°C (Fig. 2). Here, we designed the construct with a thioredoxin fusion tag, which is a highly soluble and thermostable with robust folding characteristics, therefore, enhancing the solubility of the protein (Fig. 1) (22–24). A polyHis (His_6_) tag at both N and C-terminals for affinity Ni-NTA chromatography (Fig. 1) (25).

### Functional assessment infers stable mCherry-fusion proteins

The mCherry-LA3490 protein containing 2 tandemly repeated N-terminal ricin B domains (RBL1 and RBL2) and C-terminal domain/DNase activity (CTD) was purified as a stable multidomain globular protein (Fig. 3). The fusion protein reacted with anti-His_6_ monoclonal antibodies showing that His_6_ residues were accessible for reactivity and the fusion protein was produced as in its native condition (Fig. 3d). N-terminal ricin B domains of VM proteins are rich in aromatic patches and the RBL1 domain possesses a characteristic (QxW)_3_ motif which allows them to bind to the carbohydrate moiety receptor present on the host cell surface. RBL1 domain of LA3490 binds to terminal galactose and N-acetyl-galactosamine residues such as asialofetuin, a model protein (26–28), like ricin B chain, validating the functional activity of LA3490 VM protein (Fig. 3e).

### Purification for full-length LA1402 and LA0591 (CTD) strengthens the reproducibility

**Figure 4.**
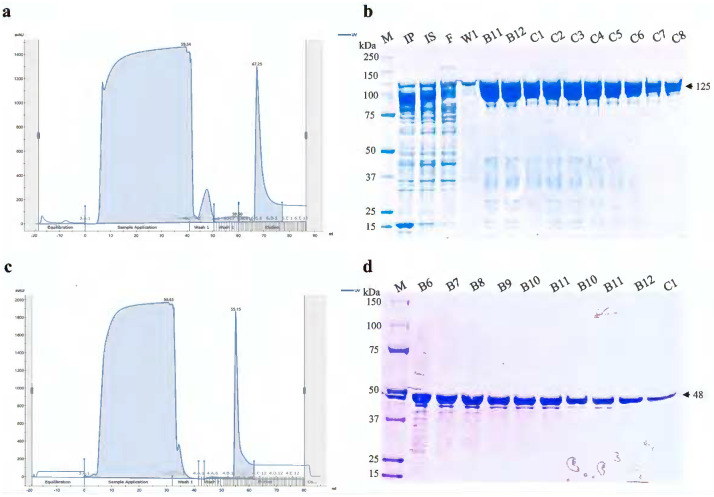
Purification of mCherry-LA1402 and tagless LA0591 using FPLC. **(a)** The chromatogram showing eluted peak is the mCherry-LA1402. **(b)** Eluted fractions were analyzed by 4–12% SDS-PAGE followed by Coomassie stain. IP, IS, F, and W1 represent induced pellet, induced supernatant, flow through, and wash respectively. B11-C8, are the eluate fractions. **(c)** LA0591, a natural variant lacking N-terminal ricin B domains was cloned without mCherry fluorescent tag. The chromatograph shows the purification of soluble LA0591 protein. **(d)** Eluted fractions were analyzed by 4–12% SDS-PAGE followed by Coomassie stain. IP, IS, F, and W1 represent induced pellet, induced supernatant, flow through, and wash respectively. B6-C1, are the eluate fractions. M represents the molecular weight marker.

To examine the reproducibility of the purification protocol, the two paralogous members LA1402 and LA0591 from the PF07598 gene family were selected to validate the purification protocol. High-level expression and successful production of mCherry-LA1402 and tagless LA0591 VM proteins verified the protocol (Fig. 4). Based on protein sequences, LA1402 show 60% identities with LA3490, and LA0591 shows 56% identities with LA3490, and they both show 99% coverage with LA3490.

### Purified soluble VM proteins are physiochemically stable

**Figure 5.**
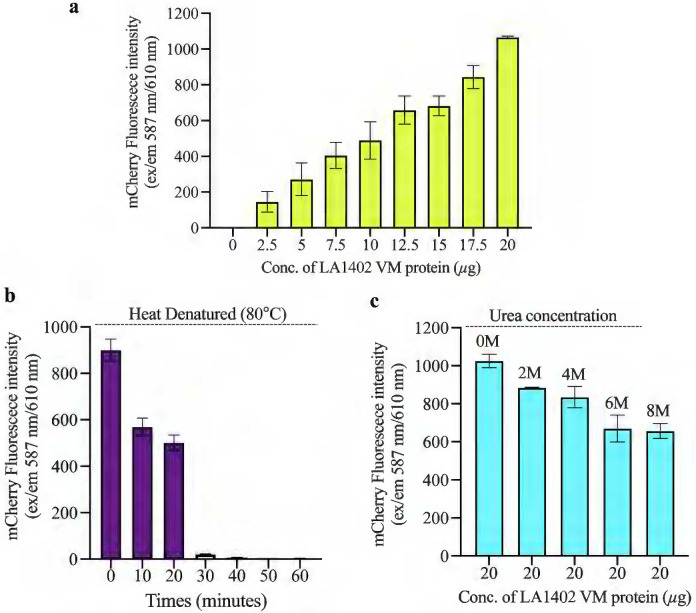
Spectrofluorimetric-based analysis of physio-chemical properties of mCherry-LA1402. CD-spectra of mCherry-LA1402 showing the secondary (helix and ß-sheet) structure content. Dose-dependent fluorescence intensity of mCherry-LA1402 was examined at ex/em 587nm/610nm. **(c)** The fusion protein was treated at 80°C at 10-minute intervals of up to 60 minutes and mCherry fluorescence intensity was determined. **(d)** The effect of urea concentration was examined on mCherry-LA1402. The gradual decrease in the intensity of mCherry-LA1402 was observed as the concentration of urea increased.

The robustness of the predicted VM protein structures was validated by examining the content of the secondary structures of mCherry-LA1402 using CD-spectroscopy (Fig. 5a, Table 1). Previously, we showed the stability of secondary structure content (*α*-helix and ß-sheets) in the paralogs LA1400 and LA0591 by CD-spectroscopy. The robustness of the CD-spectroscopy result was comparable with AlphaFold-derived 3D structures (29).

Spectrofluorometric-based analyses suggest that LA1402 is intact with mCherry and the fluorescence intensity gradually increased as the concentration of mCherry-LA1402 increased (Fig. 5b). The fusion protein was unstable at 80°C with increased time intervals (Fig. 5c). Urea had minimal effect on mCherry-LA1402 stability, even at 8M urea, under which conditions the visibly pink color and fluorescence intensity was able to be determined (Fig. 5d), suggesting the fusion protein is stable.

## Material and Methods

### Bacterial strains and reagents

MAX Efficiency^™^ DH10B Competent *E. coli* strain DH10B (Invitrogen, USA) was used for gene cloning, and strain SHuffle^®^T7 competent *E. coli* cells (New England Biolabs, USA) was used for protein expression and purification. *E. coli* was grown in Luria-Bertani (LB) medium (BD Biosciences, Sparks, MD) supplemented with 100 *μg*/mL ampicillin (Sigma-Aldrich, St. Louis, MO).

### Strategy and design of recombinant constructs

Post-signal synthetic *E. coli* codon-optimized VM genes encoding sequences *la3490* (Uniprot ID: Q8F0K3) or *la1402* (Q8F6A7) or *la0591* (Q8F8G6) linked to mCherry (X5DSL3) via a glycine-serine hinge (GGGGSGGGGSGGGGS) were synthesized and cloned into pET32b (+) (Gene Universal Inc., USA) between enterokinase cleavage sites for convenient removal of the mCherry fluorescent tag. The two histidine (His_6_) tags were added to both N and C-terminals for Nickel-NTA purification. Before use, the sequence, and the orientation of the genes in the constructs were verified by restriction digestion and sequencing (Fig.1).

### Soluble recombinant fluorescent protein expression, and purification

The cysteine-rich LA3490 or LA1402 or LA0591 VM proteins were expressed in T7 SHuffle^®^ *E. coli* cells (New England Biolabs, USA) owing to their capacity to promote disulfide bonds in the cytoplasm. Transformants were subcultured into an LB medium containing 100 *μ*g/mL ampicillin. At OD of 0.6, expression was induced at 16°C and 250 rpm for 24 h via the addition of 1 mM isopropyl-β-D-thiogalactoside (IPTG; Sigma-Aldrich, USA). Following induction, cells were pelleted by centrifugation and then lysed in CelLytic^™^ B (Cell Lysis Reagent; Sigma-Aldrich, USA) containing 50 units benzonase nuclease (Sigma-Aldrich, USA), 0.2 *μ*g/mL lysozyme, non-EDTA protease inhibitor cocktail (Roche, USA) and 1 mM PMSF (Sigma-Aldrich, USA) for 30 minutes at 37°C. Lysates were centrifuged at 4°C and 18,514 *g* for 10 min. Supernatants and pellets were separated and then analyzed by 4–12% bis-tris sodium dodecyl sulfate-polyacrylamide gel electrophoresis (SDS-PAGE). Protein concentrations were determined by BCA assay (Thermo Fisher Scientific, USA).

Recombinant thioredoxin [TRX]–His_6_–VM protein–(Gly_4_S_3_)–mCherry–His_6_ fusion proteins (using pET32 vector) were purified using a 5 mL pre-packed Ni-Sepharose AKTA Hi-TRAP column (GE Healthcare, USA) pre-equilibrated with a buffer containing 100 mM NaH_2_PO_4_ and 10 mM Tris-HCl, 25 mM imidazole, pH 8.0. Bound fusion proteins were eluted in the presence of 500 mM imidazole, pH 8.0. Eluates were pooled, concentrated via a 30 kDa Amicon^®^ Ultra centrifugal filter, then centrifuged using a high-capacity endotoxin-removal spin column (Thermo Fisher Scientific, USA) to eliminate lipopolysaccharide contamination. Recombinant protein preparations were dialyzed overnight against 1X PBS (pH 7.4) with gentle stirring (350 rpm) at 4°C (30 kDa cutoff, Slide-A-Lyzer, Thermo Scientific^™^, USA), followed by size exclusion via a 40 kDa Zeba^™^ desalting spin column (Thermo Fisher Scientific, USA) to remove salts and imidazole.

### Protein Analysis and Western blot

Purified proteins were analyzed by SDS-PAGE (30). Proteins were transferred to nitrocellulose membranes, which were then blocked for 2 h with 5% non-fat dry milk dissolved in 1X TBST buffer (AmericanBio, USA), and then probed with either mouse anti-His_6_ monoclonal antibody (1:2,000 dilution; Santa Cruz Biotechnology, USA) or mouse anti-LA3490 polyclonal antibodies (1:2,000 dilution). After washing thrice with TBST, membranes were incubated for 2½ h with alkaline phosphatase-conjugated goat anti-mouse IgG (H+L) as the secondary antibody (KPL, USA) at a dilution of 1:5000 in TBST. Blots were developed in 5-bromo-4-chloro-3-indolyl phosphate and nitroblue tetrazolium solution (BCIP/NBT; KPL, USA).

### CD-spectroscopy analysis

Circular dichroism (CD) spectroscopy of full-length LA1402 VM protein was performed using 0.2 mg/mL protein in 0.01 cm path lengths at 20°C using a Chirascan (Applied Photophysics, United Kingdom) as published (29). Briefly, LA1402 VM protein was dialyzed against 0.1 M borate buffer pH 8.5. The spectra presented as an average of triplicate scans and were recorded from 180 nm to 260 nm at a speed of 1 nm/s. The background was corrected against the buffer blank. The data were analyzed with CDNN software in-plugged with Pro-data viewer-Chirascan analyzed the CD-spectra and determined the percentage of secondary structure content.

### Quantitative analysis of mCherry tagged VM protein and their chemical and thermal stability

The fluorescence intensity of mCherry-tagged recombinant VM proteins was measured with GloMax^®^ Explorer Multimode Microplate Reader (Promega, USA) at room temperature. The reaction was performed in a final volume of 250 *μ*L of PBS containing LA1402 VM protein in the range of 20 *μ*g - 0 *μ*g in a 96-well black fluorescence-based assay plate (Thermo Scientific, USA). The excitation wavelength was set at 587 nm and the intensity of emitted fluorescence of mCherry at 610 nm was measured at the same time. The effect of urea on the fluorescence intensity of VM proteins was measured by adding gradient concentration (8M-2M) of urea in a total of 250 *μ*L reaction buffer. Simultaneously, 20 *μ*g of protein in 250 *μ*L reaction buffer were incubated at 80°C and the fluorescence intensity was measured in 10 minutes intervals for 60 minutes. The experiment was performed twice in triplicate. Graphs were generated using the software Prism version 9. Final figures were prepared using Adobe Illustrator-25.2.

### Fluorescence microscopy

Aliquots of 50 *μ*L of T7 shuffle^®^ *E. coli* culture expressing mCherry VM proteins was directly mounted onto a glass slide and air-dried. The cells were visualized using an Olympus IX81 fluorescence microscope (Tokyo, Japan). The DAPI filter (excitation/emission peak at 358/461) was used to detect nuclear signals, and the TRITC filter (excitation/emission peak at 544/570) was used to detect mCherry fluorescence. Representative images were captured using Micromanager software.

### Functional assay

Binding assays using mCherry-tagged soluble VM proteins were performed using Immulon^®^ 2HB flat-bottom microtiter plates (Thermo Fisher Scientific, USA) pre-coated with asialofetuin (5 ng/*μ*L in carbonate-bicarbonate buffer, pH 9.4) as published (19).

## DISCUSSION

Here we demonstrate the efficient production of mCherry-fusions, cysteine-rich, leptospiral VM exotoxins, both full-length (including the N-terminal ricin B domains and using a tagless construct harboring the *la0591* gene. LA0591 is a unique VM protein that lacks ricin binding domain (RBLs), therefore secreted intracellularly (18, 19). The functional assay of the mCherry-fusions was verified by immunoblotting, asialofetuin binding assay, CD-spectroscopy, and physio-chemical study. The mCherry-fusion proteins were obtained as a soluble, visibly pink color protein from the lysis-optimized protocol. Among the various detergents, CelLytic^™^ B (Cell Lysis Reagent; Sigma-Aldrich, USA) buffer showed the preeminent result over the other solubilization buffers such as 1% or 0.1% TritioX-100, 0.1% TritonX-100 with CHAPS, 2% or 5% Sarkosyl, or 8M urea (31) (Fig. 2–3, Fig. S1).

The mCherry tag had the important advantage of enabling real-time monitoring of pink color fusion protein and minimizing the time of the downstream process if the fusion protein does not turn pink color. In addition, polyhistidine tags are an excellent choice for affinity chromatography. These His_6_ tags are positioned on either the N-or C-terminals or both ends to conquer the potential problem that can occur in the inaccessibility of the protein tag, due to the obstruction from the protein folding (25, 32). Both tags successfully help in the production of fusion proteins.

VM exotoxins conform to the AB toxin paradigm. Although, their unique architecture is encoded by a single gene, they self-assembled into a multi-domain holotoxin, which differentiates them from the other classical AB toxins such as diphtheria toxin, pertussis toxin, Shiga toxin, or ricin toxin which are typically encoded by two or more genes (29). Each domain of the VM protein exotoxins shows sequence, structure, and function similarities with diverse origins (RBL1 with ricin B chain (plant origin), RBL2 with CARDS toxin from *Mycoplasma pneumoniae* (bacterial origin), and C-terminal domain with DNase (mammalian origin) respectively (19), which makes VM exotoxin a complex, all-in-one, multidomain protein.

There are various limitations in producing active proteins or toxins: 1) toxins may be toxic to *E. coli*, by causing significant defects in bacterial growth or death that dramatically decrease the expression capabilities (10, 33); and 2) cysteine-rich, proteins may be difficult to produce, because of their hydrophobicity, or the possibility of expression in inclusion bodies (34–37), although the inclusion bodies have possible advantages if they can be refolded and recovered as soluble and active forms (38, 39).

The production of inclusion bodies or aggregated proteins could be due to: 1) a high rate of protein expression formed due to specific intermolecular interaction, 2) Unbalanced protein homeostasis equilibrium, 3) the rate of heterologous recombinant protein expression surpassing the capacity of the host cells to manage protein post-translational modification, 4) environmental conditions (37, 40). However, the aggregation process is reversible (41).

To circumvent inclusion body formation, the VM gene constructs were designed in the pET32 expression vector, to be co-expressed with a thioredoxin tag in SHuffle^®^T7 competent *E. coli* cells (New England Biolabs, USA). VM protein expression was seen in both inclusion bodies and soluble fractions when recombinant protein expressed ITPG-induced at a low temperature (16°C for 24 h) (18, 19). Nonetheless, the active protein was produced from a soluble fraction which was advantageous over refolding.

The refolding of inclusion bodies into bioactive forms is extremely cumbersome, either producing low-yield or in-active proteins. The refolding potentially involves buffer exchange which reduces the protein-protein interaction and minimizes the aggregation of proteins (37, 42), which can be obtained by Size exclusion chromatography and adsorption chromatography.

Chaperonins and several additives (L-arginine, low-concentration (1–2 M) urea or guanidine hydrochloride, dithiothreitol (DTT), ß-mercaptoethanol (ß-ME), detergents, acetone, acetamide, sugar, short-chain alcohols, DMSO and PEG) have been successfully used in refolding of bioactive proteins from inclusion bodies (37, 43). They also possess an advantage in minimizing the aggregation during the crystallization (44).

The comprehensive study using advanced Raman spectroscopy and Attenuated Total Reflection-Fourier Transform Infrared (ATR-FTIR) techniques verified the functional active sites in the inclusion bodies, which was potentially possible because misfolded or aggregation-prone regions were located distantly from the active sites. This suggests that the native-like protein structure in inclusion bodies or the aggregated molecule comprised of active molecules co-existing with inactive (β-sheet-enriched) molecules instantaneously in a single aggregate unit (45–47). Hence, the structural, and functional studies of inclusion bodies are required to validate the activity of the protein, also minimize the extensive protein refolding procedures.

The functional activity of protein also depends on the sustainability of disulfide bonds. The disulfide bond formation often occurs in the periplasm, which assists various functions in structural, catalytic, and signaling pathways (40). They are formed post-translationally by the oxidation of a pair of cysteines, though the mispairing of the bonds, leads to the production of misfolded, aggregated, or insoluble inclusion bodies resulting in low yield or non-functional proteins (1, 48, 49). Although, various genetically engineered expression-host stains such as Origami and SHuffle stains have been developed by abolishing thioredoxin reductase (*trxB* gene) and glutaredoxin reductase (*gor* gene), which maintain the reducing environment in the cytoplasm and disfavor the formation of disulfide bonds (1, 40). These strains are also genetically modified for the constitutive expression of a disulfide bond isomerase, which offers an oxidative environment to promote the correction of mis-oxidized proteins (40).

SHuffle^®^T7 competent *E. coli* cells are one asset in the production of soluble proteins and have been successfully used to produce cysteine-rich VM exotoxins from *L. interrogans* and can be useful to the orthologous family in pathogenic *Leptospira* (50) as well as other cysteine-rich bacterial proteins.

In conclusion, we established the protocol to produce cysteine-rich, mCherry-fusion proteins using the Shuffle expression host, thioredoxin tag, and low temperatures for the culture growth. The mCherry-fluorescent tag added sophistication for real-time detection of the fusion protein and advantage in various functional studies.

## Supplementary Material

1 Figure S1. Optimization of expression and purification of mCherry-LA3490
The pellet of clone expressing mCherry-LA3490 was solubilized using various approaches, and the purification was performed using the standard protocol mentioned in the experimental procedures. **(a)** Lane 1 shows induced supernatant which was obtained upon solubilization in CelLytic^™^ B Cell Lysis Reagent. Lane 2 shows induced supernatant solubilized in lysis buffer containing 20 mM Tris-HCl + 150 mM NaCl + 1% TritonX-100. Lanes 3- and 4 show induced supernatant solubilized in CelLytic^™^ B Cell Lysis Reagent containing 1% and 0.01 % TritonX-100 respectively. Induced soluble fraction solubilized in CelLytic^™^ B Cell Lysis Reagent containing 0.01 % TritonX-100, was used to purify the LA3490 proteins. The eluted fractions were analyzed on 4–12% SDS-PAGE (right panel). F and W1–3 represent flow through and washes. E1-E5 is the eluate fractions. **(b)** The recombinant clone of mCherry-LA3490 producing pellet was solubilized in CelLytic^™^ B Cell Lysis Reagent containing 0.01 % TritonX-100 and 1 mM CHAPS. IP and IS represent induced pellet and induced supernatant. F and W1–2 represent flow through and wash. E1-E9 is the eluate fractions. **(c)** The pellet was solubilized in lysis buffer 100 mM NaH_2_PO_4_, 10 mM Tris-HCl pH 7.4 containing 8M urea followed by sonication and separation of the pellet (IP) and supernatant (IS) fractions. In addition, the pellet was solubilized in lysis buffer 20 mM Tris-HCl + 150 mM NaCl containing 2% or 5 % sarkosyl. The induced soluble fraction solubilized with 5% sarkosyl was subjected to purification using AKTA pure and eluted fractions were analyzed on 4–12% SDS-PAGE (right panel).

## Figures and Tables

**Table 1.** Assessment of stability of the secondary structure of soluble VM protein

LA1402	200–260 nrn	205–260 nm	210–260 nm
Helix	37 %	32.7 %	32 %
Antiparallel	8.8 %	8.1 %	8.6 %
Parallel	8.9 %	9.2 %	9.0 %
ß-turn	17.0 %	16.9 %	17.1 %
Random coil	32.7 %	33.7 %	34.0 %
Total Sum	100.2 %	100.5 %	100.8 %

## Data Availability

The datasets used and analyzed during the current study are available from the corresponding author upon reasonable request.
