# Supplementary material for: mCherry Fusion Proteins Facilitate Production of Recombinant, Cysteine-Rich Leptospira interrogans Proteins in Escherichia coli
Source: Res Sq. 2023 May 18:rs.3.rs-2931251. Preprint. [Version 1] doi: 10.21203/rs.3.rs-2931251/v1 (PMC10246097; doi:10.21203/rs.3.rs-2931251/v1)

Fig. Supplemental

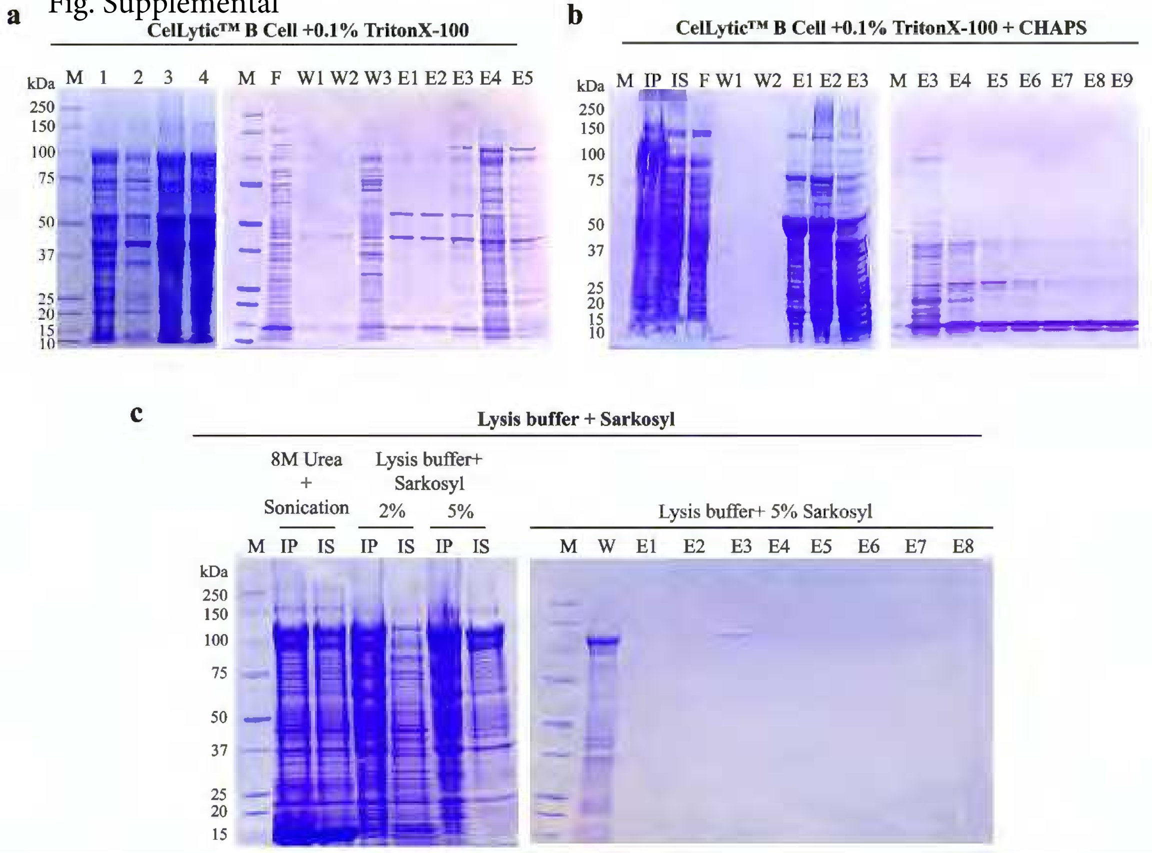

Supplement: 1 — Figure S1. Optimization of expression and purification of mCherry-LA3490 The pellet of clone expressing mCherry-LA3490 was solubilized using various approaches, and the purification was performed using the standard protocol mentioned in the experimental procedures. (a) Lane 1 shows induced supernatant which was obtained upon solubilization in CelLytic™ B Cell Lysis Reagent. Lane 2 shows induced supernatant solubilized in lysis buffer containing 20 mM Tris-HCl + 150 mM NaCl + 1% TritonX-100. Lanes 3- and 4 show induced supernatant solubilized in CelLytic™ B Cell Lysis Reagent containing 1% and 0.01 % TritonX-100 respectively. Induced soluble fraction solubilized in CelLytic™ B Cell Lysis Reagent containing 0.01 % TritonX-100, was used to purify the LA3490 proteins. The eluted fractions were analyzed on 4–12% SDS-PAGE (right panel). F and W1–3 represent flow through and washes. E1-E5 is the eluate fractions. (b) The recombinant clone of mCherry-LA3490 producing pellet was solubilized in CelLytic™ B Cell Lysis Reagent containing 0.01 % TritonX-100 and 1 mM CHAPS. IP and IS represent induced pellet and induced supernatant. F and W1–2 represent flow through and wash. E1-E9 is the eluate fractions. (c) The pellet was solubilized in lysis buffer 100 mM NaH2PO4, 10 mM Tris-HCl pH 7.4 containing 8M urea followed by sonication and separation of the pellet (IP) and supernatant (IS) fractions. In addition, the pellet was solubilized in lysis buffer 20 mM Tris-HCl + 150 mM NaCl containing 2% or 5 % sarkosyl. The induced soluble fraction solubilized with 5% sarkosyl was subjected to purification using AKTA pure and eluted fractions were analyzed on 4–12% SDS-PAGE (right panel). [file NIHPPRS2931251V1-supplement-1.pdf]
